# A Conversation with Rob Jackson

**DOI:** 10.1021/acscentsci.4c01007

**Published:** 2024-07-01

**Authors:** Katherine Bourzac

Climate change is scary. But Stanford University climate scientist Rob Jackson says this should motivate us to take action. In his new book, Into the Clear Blue Sky, Jackson takes readers on a world tour of climate solutions. Jackson introduces scientists and entrepreneurs who are developing green steel, plant-based meat, and carbon sequestration technologies, and local activists who are restoring wetlands and advocating for climate justice.

**Figure:**
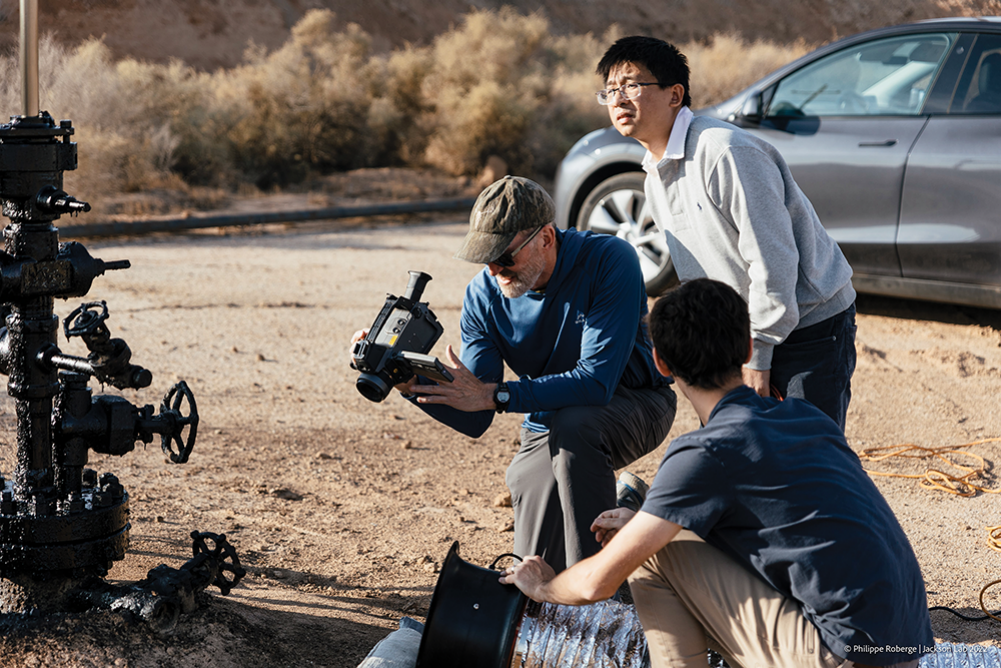
Rob Jackson (center, holding device) imaging and measuring methane leaks from an oil and gas well. Credit: Philippe Roberge/Stanford University.

Jackson has a front-row seat to the world’s still-rising greenhouse gas emissions. He’s chair of the Global Carbon Project, a group of hundreds of volunteer scientists who calculate and publish what he calls a “pulse-of-the-planet estimate” of emissions.

The atmosphere is in need of repair. But Jackson is particularly optimistic about the potential for restoring atmospheric methane to preindustrial levels—something he says we can accomplish in our lifetimes if we start cutting emissions now. Katherine Bourzac talked with Jackson about his book, his research on methane, and how we can, as he puts it, go “from climate despair to climate repair.” This interview was edited for length and clarity.

## What inspired you to write this book?

I wanted to try and reach an audience beyond the people I normally do.

I view my book as a home repair manual for the planet. It highlights the people and the ideas needed to solve the climate crisis. I want most of all to give people hope, a sense of optimism. Yes, climate change is already bad, but we can still fix this problem.

## Where are we now with greenhouse gas emissions?

Concentrations of carbon dioxide, methane, and nitrous oxide are all at record highs. None of them have peaked, let alone started to drop.

That sounds more discouraging than it is. There’s a lot happening in clean energy, especially. The solar panels and the wind turbines are helping a lot, but we’re not reducing energy use. We’re using solar to address new energy demand rather than to take fossil energy offline. We need renewables to displace fossils, not just add to new energy demands.

## What about the targets set by the Paris Agreement, which commits to limiting global warming to 1.5 or 2 °C over preindustrial levels? Are we on track, and have temperature increases been a helpful way to talk about climate change?

We’ve blown by the 1.5 °C temperature threshold, and we’re sprinting toward 2 °C. Obviously, temperature thresholds are important, but I’m trying to provide a narrative that resonates with people.

In this book I try to use the idea of restoration as a way of resetting the narrative on climate action. The Endangered Species Act requires us to bring endangered species back to health, not just to keep them alive. When we see grizzly bears in Yellowstone meadows, when we see gray whales migrating to Alaska each spring, we’re seeing the planet restored. Our goal for the atmosphere should be the same.

## Do you think that we’re at the point where we need not only to reduce emissions but also to remove greenhouse gases from the atmosphere?

For years, I viewed greenhouse gas removal or negative-emission technologies as a distraction. They can be used to delay activity and mitigation today if we think, “Oh, don’t worry, we’ll just pull them out of the air tomorrow, or next century.”

But we have gone so long without cutting emissions that we now need both. Mitigation first, always. And we’re going to need to pull some greenhouse gases out of the air, I believe, to keep a habitable planet.

But realistically, it may not be possible. Why should we expect people to spend trillions of dollars to pull carbon dioxide out of the air in the next decade or in the next generation when we weren’t willing to spend billions of dollars to keep it out of the air today?

**Figure:**
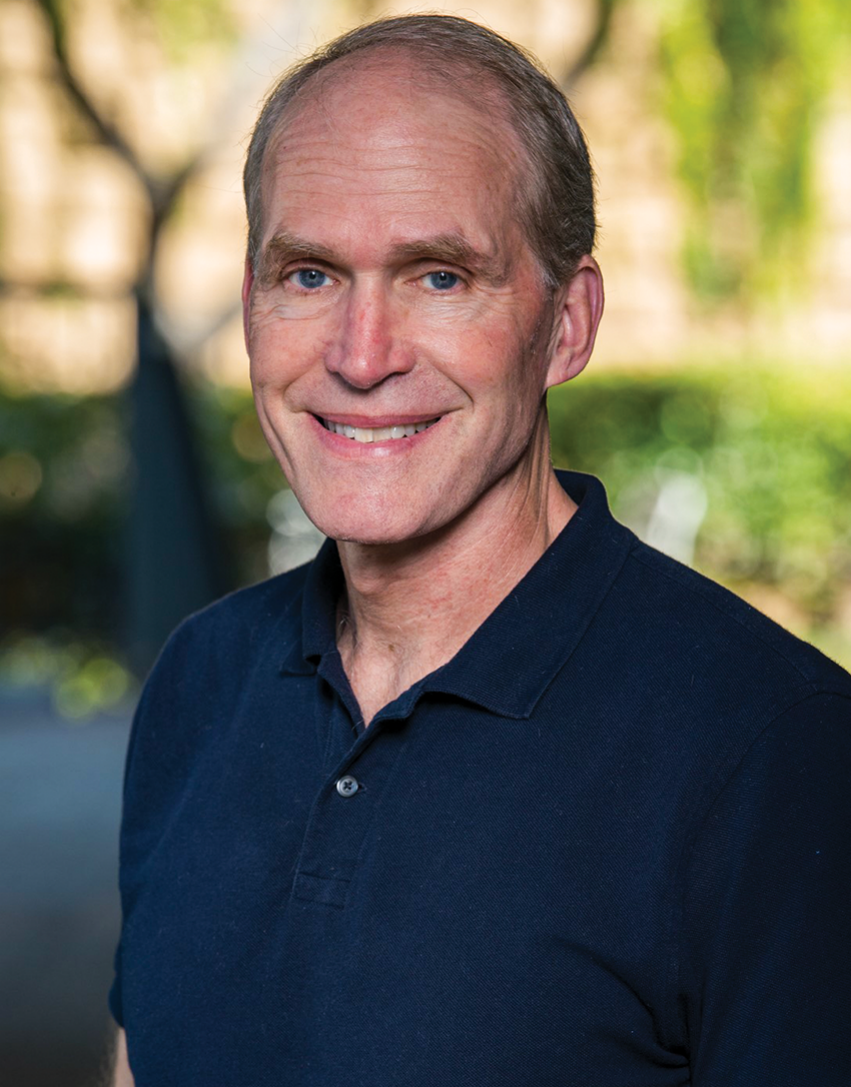
Credit: Stanford Doerr School of Sustainability.

## What can individuals do?

Transportation and our homes are what we most control.

Electric vehicles will ultimately win out over gas combustion because they’re faster and better and require less maintenance. That transition will take decades, though. The biggest source of emissions for most readers is probably flying. So flying less is something we can all do. The backdrop to that is that most of the people on Earth have never flown.

We can also control whether we use gas or electricity at home. I spent the last decade documenting the climate and health benefits of moving from gas to electric appliances. Our stoves emit methane, a potent greenhouse gas; benzene, a carcinogen; and nitrogen oxide gases, which are an asthma trigger.

## Why have you shifted your research focus to methane in recent years?

Methane has warming superpowers. It is more potent than carbon dioxide, and shorter lived. If we stopped emitting methane today with a magic wand, the atmosphere would return to normal within 10 or 20 years. We would save half a degree Celsius of warming by doing so. There’s no other lever we can pull to have so much influence on the climate in the short term.

If we stop emitting carbon dioxide today, there would still be a trillion extra tons of it in the air a century or 1,000 years from now. Restoring the atmosphere for carbon dioxide and nitrous oxide is something I will never see. But I do dream of seeing it for methane.

## In the book you discuss how the level of consumption in the US drives our outsize carbon emissions. Is climate change primarily a technology problem, or do we need to consume less?

Yeah, it’s a question that isn’t asked often enough. I tell students I don’t believe we can build our way out of climate change. We do need technological solutions. But we have to use less, certainly in the richest countries like the US.

We have almost a car per person in the US. If everyone on Earth owned a car, there would be 7 billion cars. I don’t care whether they’re EVs or hydrogen cars, the world would not be better off with 5 or 6 billion more vehicles.

It’s easier to talk about the next green technology than it is to say, “What if we just didn’t build that?” If we took one coal or gas power plant offline, it would keep far more carbon dioxide from the air than all the direct-air-capture capacity that’s ever been built.

**Figure:**
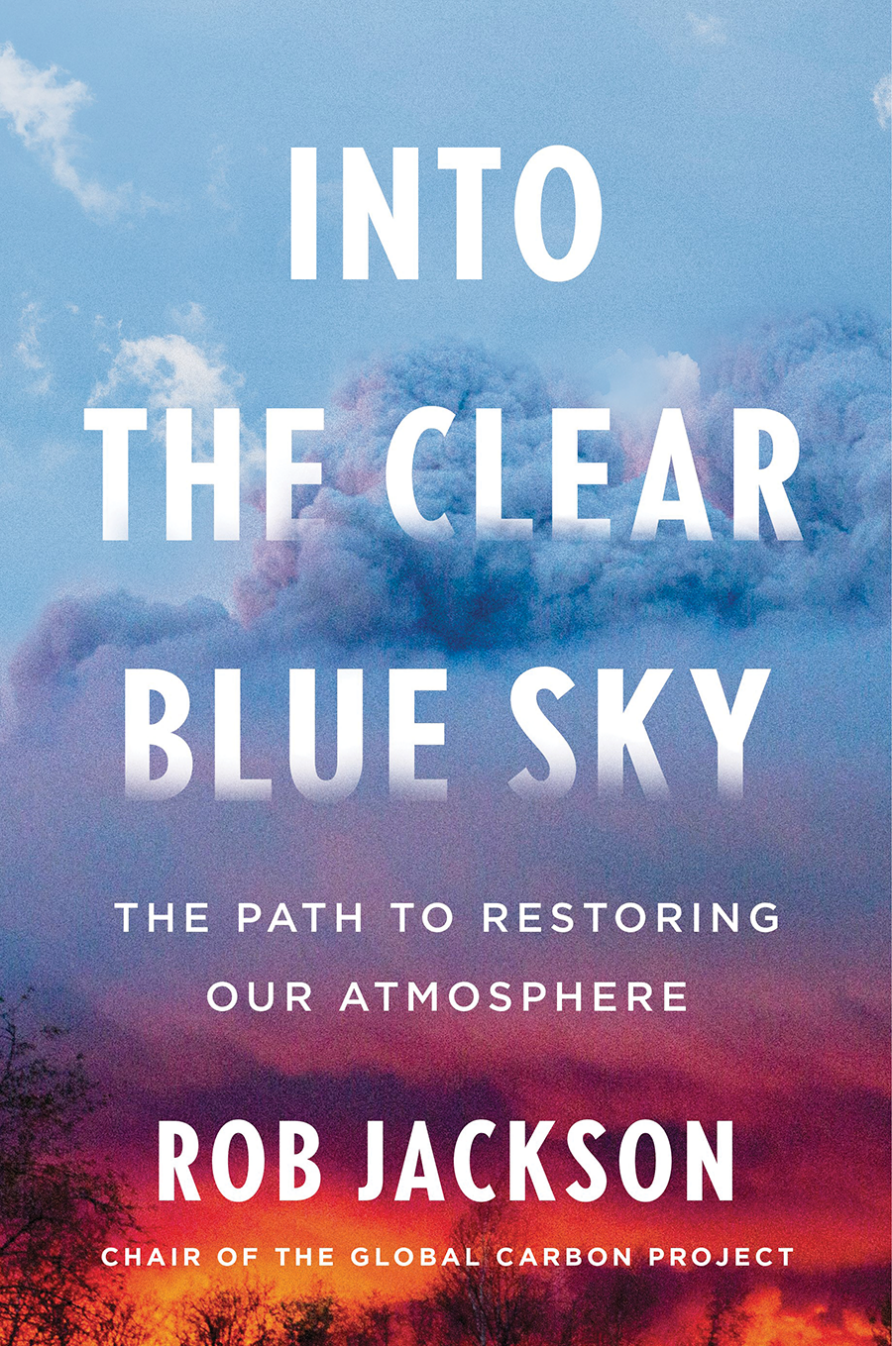
Credit: Simon & Schuster.

## You start one of the chapters in your book with the question, “What would you do if you thought we were in danger of ending civilization on Earth?” Do you think we are in danger of that?

Unchecked, I think we are. But on the other hand, air pollution and toxics in the US are a lot better than when I was a kid.

The elimination of lead in gasoline has saved billions of dollars and countless lives. We do act sometimes when the evidence is clear enough and interest groups don’t get in the way.

*Katherine Bourzac is a freelance contributor to*Chemical & Engineering News*, an independent news publication of the American Chemical Society.*

